# Impaired glymphatic system function and its association with speech and language delay in children with ADHD: a prospective study

**DOI:** 10.3389/fnhum.2025.1612997

**Published:** 2025-08-18

**Authors:** Yangchen Li, Jianhong Wang, Xinyu Yuan, Lili Zhang, Yuchun Yan, Yang Yang, Lin Wang

**Affiliations:** ^1^Department of Radiology, Capital Center for Children’s Health, Capital Medical University, Beijing, China; ^2^China-Japan Friendship Hospital, Chinese Academy of Medical Sciences & Peking Union Medical College, Beijing, China; ^3^Center of Children’s Healthcare, Capital Center for Children’s Health, Capital Medical University, Beijing, China; ^4^Chinese Academy of Medical Sciences & Peking Union Medical College, Beijing, China; ^5^Department of Medical Engineering, Capital Center for Children’s Health, Capital Medical University, Beijing, China

**Keywords:** ADHD, glymphatic system function, speech and language delay, DTI-ALPS, comorbidity

## Abstract

**Objective:**

The glymphatic system, a glial cell-dependent waste clearance pathway in the brain, is essential for the maintenance of brain homeostasis. This study aimed to explore the relationship between attention-deficit/hyperactivity disorder (ADHD) and its co-occurring clinical phenomena, including gross motor and language development, and the glymphatic system.

**Methods:**

A total of 56 children with ADHD and 33 age-and gender-matched typically developing (TD) children were included in this prospective study. Diffusion tensor image analysis along the perivascular space (DTI-ALPS) was used to calculate the ALPS index, which evaluates glymphatic system function. The ALPS index of ADHD patients was compared with that of TD, and the correlation between the gross motor retardation and speech and language delay in ADHD patients and ALPS index was further analyzed.

**Results:**

The ALPS index in ADHD patients was significantly lower than that in TD children (1.503 ± 0.153 vs. 1.591 ± 0.152, *p* < 0.05). After adjusting for age and sex, the ALPS index was negatively correlated with speech and language delay in ADHD patients (*r* = −0.329, *p* = 0.015). However, no significant correlation was found between the ALPS index and gross motor retardation in ADHD patients.

**Conclusion:**

Glymphatic system function may be impaired in ADHD patients. This study is the first to demonstrate that speech and language delay in ADHD patients may be associated with impaired glymphatic system function. Early intervention may be linked to better language trajectories in ADHD, providing a rationale for longitudinal trials to test causality.

## Introduction

Attention-deficit/hyperactivity disorder (ADHD) is characterized by significant difficulties in inattention, distractibility, and hyperactivity, leading to substantial impairments in school, home, and social functioning. These difficulties often persist into adulthood, creating a heavy burden for families and society (Thapar and Cooper, 2016). ADHD is one of the most common neurodevelopmental disorders in children, affecting approximately 5–7% of the pediatric population (Willcutt, 2012; American Psychiatric Association, 2013). However, the biological mechanisms underlying ADHD neurodevelopment remain unclear, and there are currently no diagnostic neurobiological markers.

The glymphatic system is glial cell-dependent waste clearance pathway in the brain that is essential for the maintenance of brain homeostasis (Benveniste et al., 2019; Mestre et al., 2020; Jessen et al., 2015). This system comprises a perivascular unit consisting of the vasculature, perivascular spaces (PVS), and astrocytes (Nedergaard and Goldman, 2016). Cerebrospinal fluid (CSF) enters the PVS surrounding arteries, moves through the brain parenchyma, and then drains into the PVS surrounding veins, ultimately exiting via the venous system. Toxic proteins and metabolites are cleared from the brain through this pathway (Iliff et al., 2012). Impaired glymphatic system function, resulting in poor clearance of toxic proteins and metabolites, has been implicated in the etiology of various neurological diseases. Previous studies have also supported the involvement of the glymphatic pathway in conditions such as autism spectrum disorder (ASD) (Li et al., 2022). However, research on the glymphatic system function in children with ADHD is limited.

A variety of methods can be used to evaluate the glymphatic system function, but non-invasive methods are particularly necessary for pediatric populations (Rasmussen et al., 2018). In this study, we utilized diffusion tensor image analysis along the perivascular space (DTI-ALPS), a non-invasive method with shorter scanning times compared to intravenous-contrast MRI. This method assesses the movement of water molecules along the PVS by measuring diffusivity using the diffusion tensor method. The medullary arteries and veins, which accompany the PVS, serve as the major drainage pathway of the glymphatic system. At the level of the lateral ventricle body, the medullary vein is perpendicular to the ventricle wall. Since the major fiber tracts are not parallel to the direction of the PVS, the analysis of diffusion along the perivascular space is almost independent of the major fiber tracts. When changes in diffusivity in the right–left direction (*x*-axis) are observed in both the projection and association fiber areas, these changes are attributed to alterations in diffusivity corresponding to the direction of the PVS. The activity of the glymphatic system is assessed using the ALPS index, which is determined by the ratio of two sets of diffusivity values perpendicular to the main fibers in the tissue. This ratio reflects the influence of water diffusion along the PVS, indicating glymphatic system activity (Taoka and Naganawa, 2020a).

ADHD is highly comorbid with other neurodevelopmental disorders, including communication and specific learning or motor disorders (Jensen and Steinhausen, 2015). Co-occurring ADHD and communication disorders pose a significant challenge for school-based practitioners. Previous research has suggested that up to one-third of children with ADHD may have specific language impairments (Mueller and Tomblin, 2012). Additionally, children with ADHD often exhibit motor skills problems and deficits in gross motor tasks (Goulardins et al., 2017). Several pediatric studies have also reported associations between increased extra-axial cerebrospinal fluid (EA-CSF) and motor delays (Sahar, 1978; Nickel and Gallenstein, 1987; Lorch et al., 2004; Hellbusch, 2007).

An increasing number of studies have used the DTI-ALPS method to elucidate glymphatic system function in various neurological disorders, such as dementia (Nedergaard and Goldman, 2020), idiopathic normal pressure hydrocephalus (Bae et al., 2021), traumatic brain injury (Li et al., 2020), and ischemic stroke (Gaberel et al., 2014). However, research on glymphatic system function in ADHD patients remains limited (Zhang et al., 2024). Although the clinical significance of gross motor and language development has long been recognized (Chess, 1974), the implications of the co-occurrence of ADHD with gross motor and language development are still poorly understood. No studies have further explored the relationship between glymphatic system function and complications in ADHD patients. The purpose of the present study was to examine in more details the clinical phenomenology and possible pathologic mechanisms of co-occurring ADHD and gross motor and language development.

## Method

### Study population

This prospective study recruited 63 patients with ADHD and 34 typically developing (TD) children matched by age and gender between 2022 and 2024 from the Center of Children’s Healthcare, Capital Center for Children’s Health, Capital Medical University. All participants were right-handed. ADHD diagnoses were confirmed according to the criteria of the Diagnostic and Statistical Manual of Mental Disorders, 5th edition (American Psychiatric Association, 2013). Controls were screened by the same physicians to ensure the absence of major neurologic or psychiatric disorders, as well as the absence of psychiatric disorders in their first-degree relatives. None of the controls were using any psychoactive medications.

The inclusion criteria were as follows: (1) clinically diagnosed, treatment-naïve ADHD patients or TD children without ADHD; (2) aged 6 to 14 years; (3) the child’s guardian was able to provide detailed information on the child’s gross motor and language development as well as a standardized health handbooks over an extended period. The exclusion criteria included: (1) history of seizures and/or autism spectrum disorder (ASD); (2) presence of hearing, vision, or intellectual developmental disorders (Wechsler Intelligence Scale scores <70); (3) history of brain injury or other psychiatric or neurological disorders; (4) MRI contraindications; (5) MRI data with scanning artifacts or motion artifacts; (6) MRI showing organic lesions.

A total of 56 participants with ADHD and 33 age-and gender-matched TD children were included in the study. The study protocol was approved by the hospital’s human ethics committee (NO. SHERLL2024074), and written informed consent was obtained from all participants for the examination.

### Clinical assessment

We used the Conners Parent Rating Scale to assess behavioral symptoms in children. These behaviors are grouped into conduct problems, study problems, psychosomatic problems, impulsive–hyperactive behavior, anxiety, and hyperactivity index (Goyette et al., 1978). Higher scores indicate more severe problems.

Gross motor and language development were evaluated using physician-annotated milestone documentation in standardized child health handbooks. Gross motor development was assessed by observing key milestones such as looking up, rolling over, sitting, climbing, standing, walking alone, climbing steps, and jumping with both feet. Children with gross motor function scores more than two standard deviations below the gender- and age-matched norm were identified as having gross motor retardation. Speech and language development were assessed based on speech and language developmental milestones (Coplan, 1985). Normal speech progresses through stages of cooing, babbling, words, and word combinations, while normal language progresses through stages of understanding and expressing more complex concepts. Previous studies have recommended using the ability to achieve milestones with a 75% probability as an early warning threshold (National Institute on Deafness and Other Communication Disorders, 2022). In other words, if a child does not achieve a milestone that 75% of children of the same age can achieve, it is defined as delayed speech and language development.

### MRI sequences

All MRI examinations were performed using a 3-Tesla MRI scanner (Ingenia; Philips Healthcare, Best, The Netherlands) with a 15-channel phased-array head coil. All subjects underwent the MR scan without sedation. The subject’s head was stabilized with a foam pillow to minimize motion artifacts. Axial T2-weighted sequences were collected to rule out any cranial organic lesions. A three-dimensional Turbo Fast Echo (TFE) sequence with 1.00 mm slice thickness was performed for each subject.

Diffusion tensor imaging (DTI) scans were obtained using a single-shot echo planar imaging sequence. The images were collected parallel to the anteroposterior commissure (AC-PC) in the axial direction to cover the whole brain. The scanning parameters were as follows: repetition time (TR)/echo time (TE) = 2,880 ms/89 ms, field of view (FOV) = 210 × 210 × 136 mm^3^, matrix = 128 × 128 × 68. The acquisition voxel volume was 2 × 2 × 2 mm^3^, number of excitations (NEX) = 1, number of directions = 32, 68 slices with no gap, and B value = 1,000 s/mm^2^.

### Calculation of the diffusivities and ALPS index

We used FMRIB Software Library version 6.0 (FSL; Oxford Centre for Functional MRI of the Brain, Oxford, UK; www.fmrib.ox.ac.uk/fsl) (Jenkinson et al., 2012) for processing of DTI data.

First, brain regions were extracted using the FMRIB Software Library Brain Extraction Tool. Eddy current correction was then performed for all DTI scans. The software generated diffusion tensor computational images, including color-coded fractional anisotropy maps and diffusivity maps. Diffusivity maps were obtained in the directions of the *x*-axis (right–left; Dxx), *y*-axis (anterior–posterior; Dyy), and *z*-axis (inferior–superior; Dzz) (Yokota et al., 2019).

**Figure 1:**
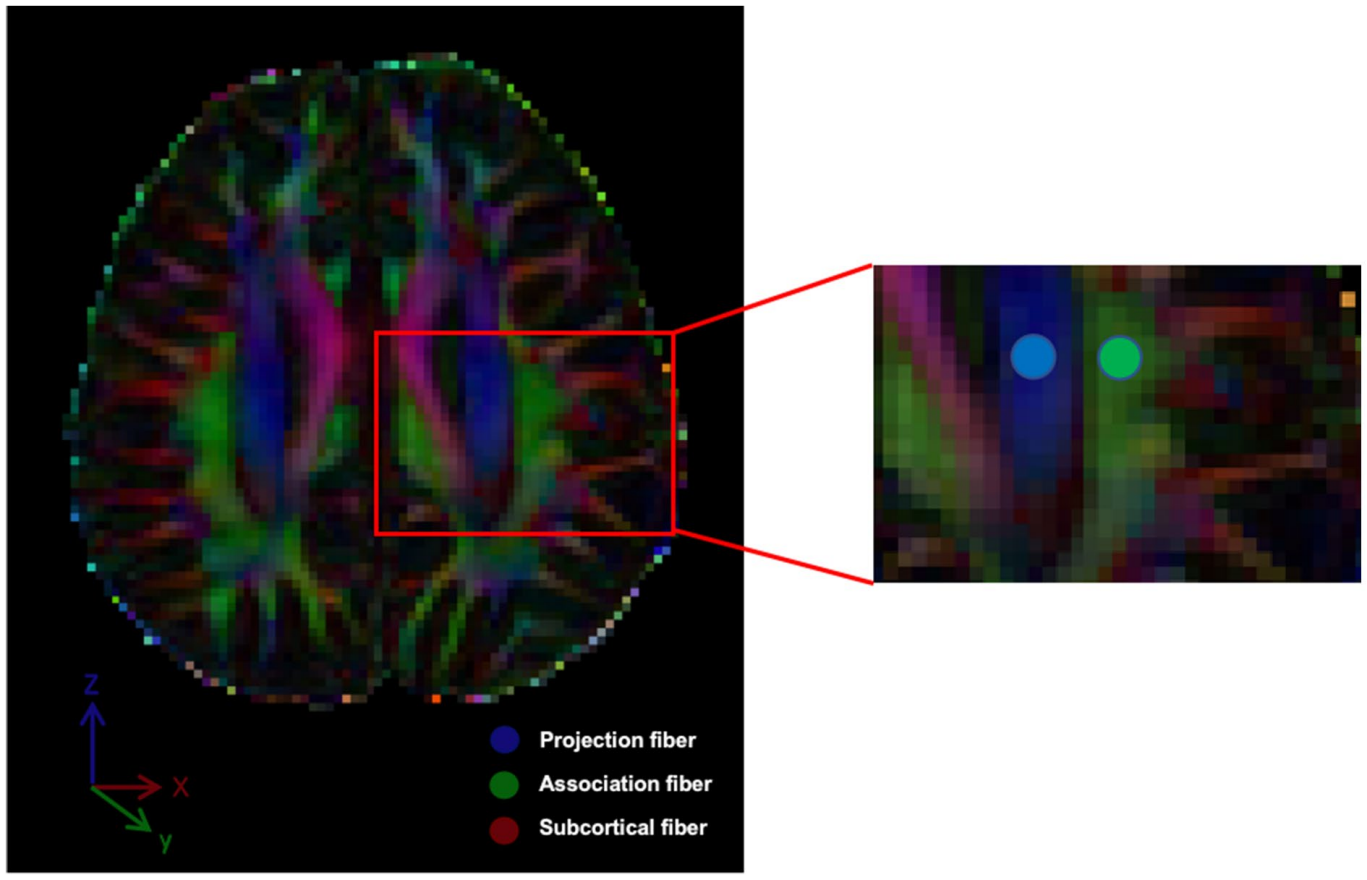
The DTI color-coded fractional anisotropy (FA) map illustrates the directions of the projection fibers (blue; *z*-axis), association fibers (green; *y*-axis) and subcortical fiber (red; *x*-axis). Two regions of interest (ROIs) were defined to measure diffusivities in these areas.

Using the color-coded FA map, we employed the approach, which has been followed by numerous papers, of manually placing circular ROIs in the left projection and association fiber areas at the level of the lateral ventricle body (Figure 1), and when setting the ROI in the area of association fibers, we avoid mixing of subcortical fibers whose main axis is in the *x*-direction. Moreover, since association fibers are often thicker on the dominant side (left side), placement of a ROI on the left side has the advantage that a stable ROI can be set, even if it is done manually (Taoka et al., 2024). The perivascular space lies perpendicular to the ventricle wall at this level and typically runs in the right–left direction (*x*-axis) on the axial plane. The *x*-, *y*-, and *z*-axis diffusivity values were measured in these ROIs, and the ALPS index was calculated for each case. The ALPS index is the ratio of the mean *x*-axis diffusivity in the projection area (Dxxproj) and the *x*-axis diffusivity in the association area (Dxxassoc) to the mean *y*-axis diffusivity in the projection area (Dyyproj) and the *z*-axis diffusivity in the association area (Dzzassoc):


$$\begin{array}{l} \text{ALPS index}=(\text{mean}(\text{Dxxproj},\text{Dxxassoc}))/\\ (\text{mean}(\text{Dyyproj},\text{Dzzassoc})). \end{array}$$

The measured values selected in this study are all on the left side.

### Statistical analysis

Statistical analyses were conducted using SPSS 25.0 (IBM Corp., Armonk, NY, USA). Demographic data between the ADHD and TD groups were compared using two-tailed independent-sample t-tests and chi-square tests. Pearson’s correlation analysis was employed to assess the association between the ALPS index and Conners total scores. Additionally, Pearson and partial correlation analyses were used to evaluate the relationship between the ALPS index and both gross motor retardation and speech and language delay. A *p*-value of less than 0.05 was considered statistically significant. Intraobserver and interobserver consistency were assessed using the intraclass correlation coefficient (ICC). An ICC value of less than 0.40 indicated poor consistency, an ICC value between 0.40 and 0.75 indicated moderate consistency, and an ICC value greater than 0.75 indicated good consistency.

## Results

**Figure 2:**
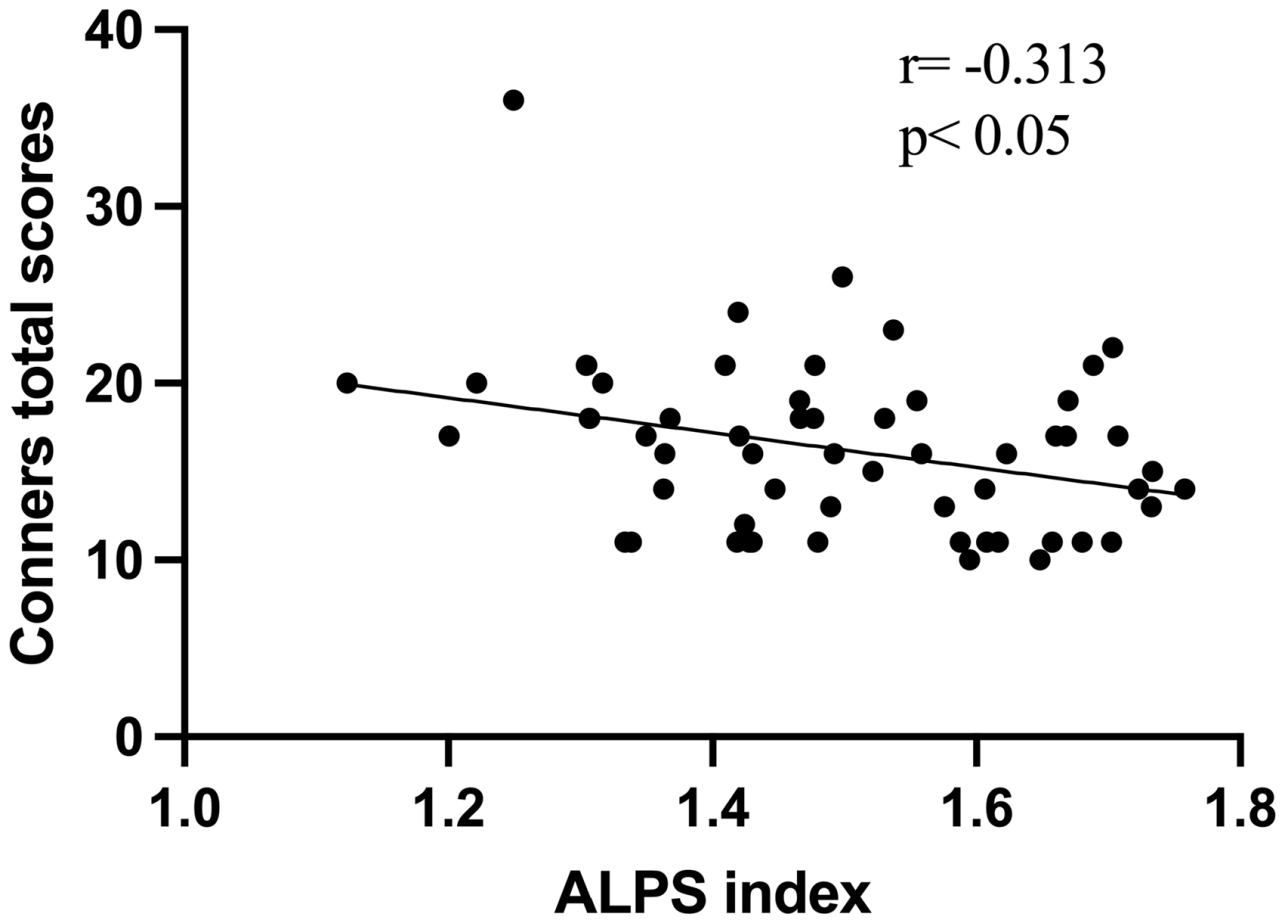
The scatter plot illustrates a negative correlation between the ALPS index and Conners total scores in 56 ADHD patients.

A total of 56 children with ADHD and 33 TD volunteers, with a mean age of 8 years, were enrolled in this study. As shown in Table 1, there were no significant differences in age or sex distribution between the ADHD and TD groups (*p* > 0.05). All participants were treatment-naïve, meaning they had not received any pharmacological or behavioral interventions. A negative correlation was observed between the ALPS index and Conners total scores (*r* = −0.313, *p* < 0.05) (Figure 2).

**Table 1:** Demographic data of ADHD and TD in the study.

Demographic Indicators	ADHD (*n* = 56)	TD (*n* = 33)	*p* value
Age	8.39 ± 2.07	8.85 ± 2.54	0.359
Male/female (*n*)	43/13	24/9	0.672
Conners total scores	16.20 ± 4.83	/	/
gross motor retardation (*n*)	4	/	/
speech and language delay (*n*)	9	/	/

**Figure 3:**
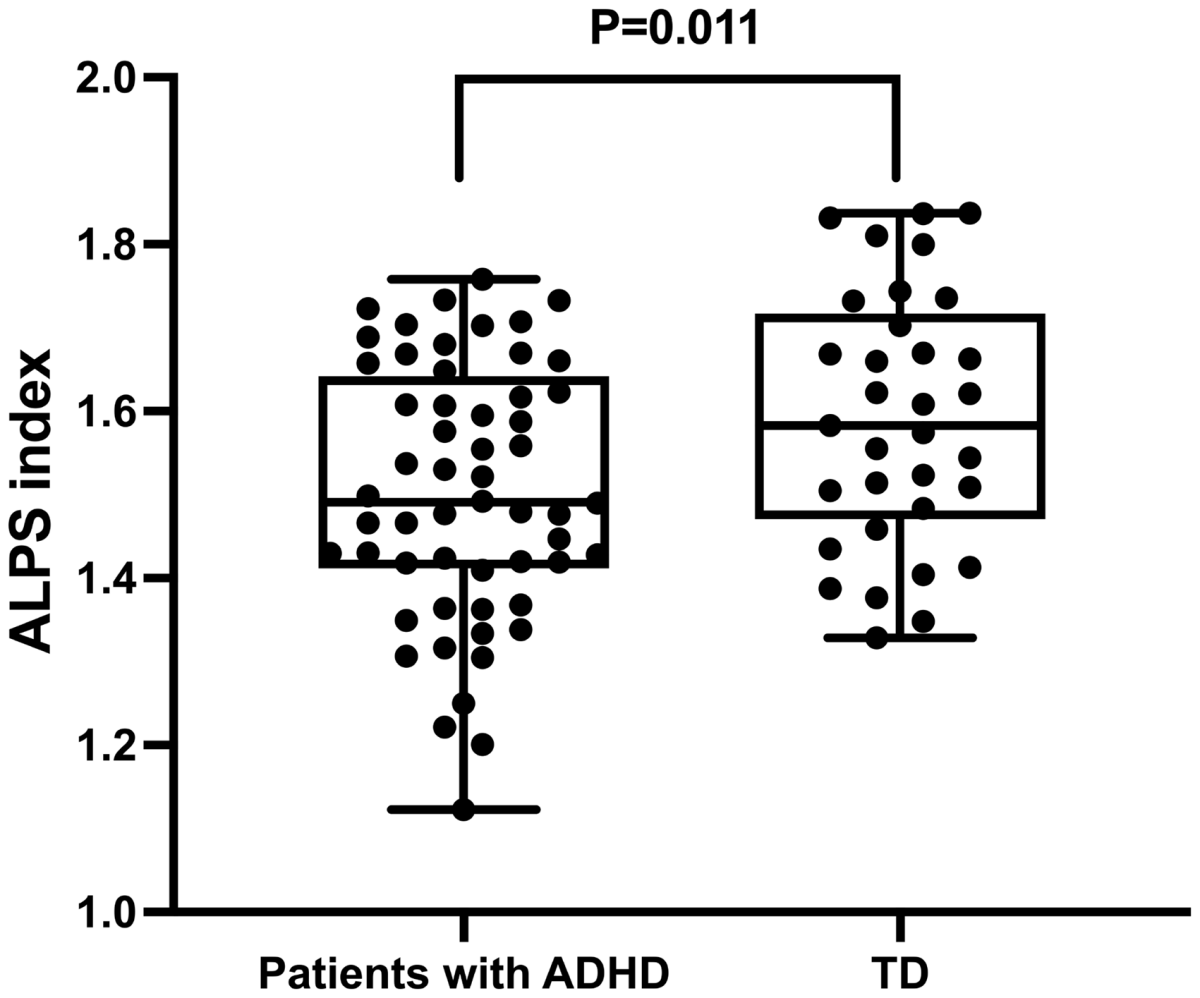
Differences in glymphatic system function between patients with ADHD and typically developing (TD) individuals. The figure demonstrates that the ALPS index in ADHD patients is significantly lower than that in TD individuals, indicating impaired glymphatic system function in ADHD.

Significant differences in the ALPS index were observed between ADHD patients and TD volunteers (Table 2). Specifically, the ALPS index was lower in ADHD patients compared to TD controls (1.503 ± 0.153 vs. 1.591 ± 0.152, *p* < 0.05) (Figure 3). Post-hoc power analysis using G*Power 3.1 revealed the study had 76% power (*α* = 0.05, two-tailed) to detect the observed group difference in ALPS indices (Cohen’s d = 0.59), indicating moderate statistical robustness. No significant differences were found between the two groups in terms of Dxproj, Dyproj, and Dxassoc (*p* > 0.05), but a significant difference was observed in Dzassoc (*p* = 0.007).

**Table 2:** Shows left diffusion tensor image analysis along the perivascular space (DTI-ALPS) index among ADHD and TD participants.

Diffusion Tensor Image Metrics	ADHD (*M* ± SD)	TD (*M* ± SD)	*p* value	Cohen’s d
D_xproj_ (×10^−3^ mm^2^/s)	0.650 ± 0.066	0.625 ± 0.087	0.122	0.32
D_yproj_ (× 10^−3^ mm^2^/s)	0.476 ± 0.072	0.453 ± 0.077	0.171	0.31
D_xassoc_ (× 10^−3^ mm2/s)	0.719 ± 0.123	0.708 ± 0.131	0.707	0.09
D_zassoc_ (×10^−3^ mm^2^/s)	0.443 ± 0.088	0.391 ± 0.080	0.007	0.62
ALPS index	1.503 ± 0.153	1.591 ± 0.152	0.011	0.59

Dxproj, diffusivity along the *x*-axis in projection fiber area; Dyproj, diffusivity along the *y*-axis in projection fiber area; Dxassoc, diffusivity along the *x*-axis in association fiber area; Dzassoc, diffusivity along the *z*-axis in association fiber area; *p* value less than 0.05 indicate statistical significance.

**Figure 4:**
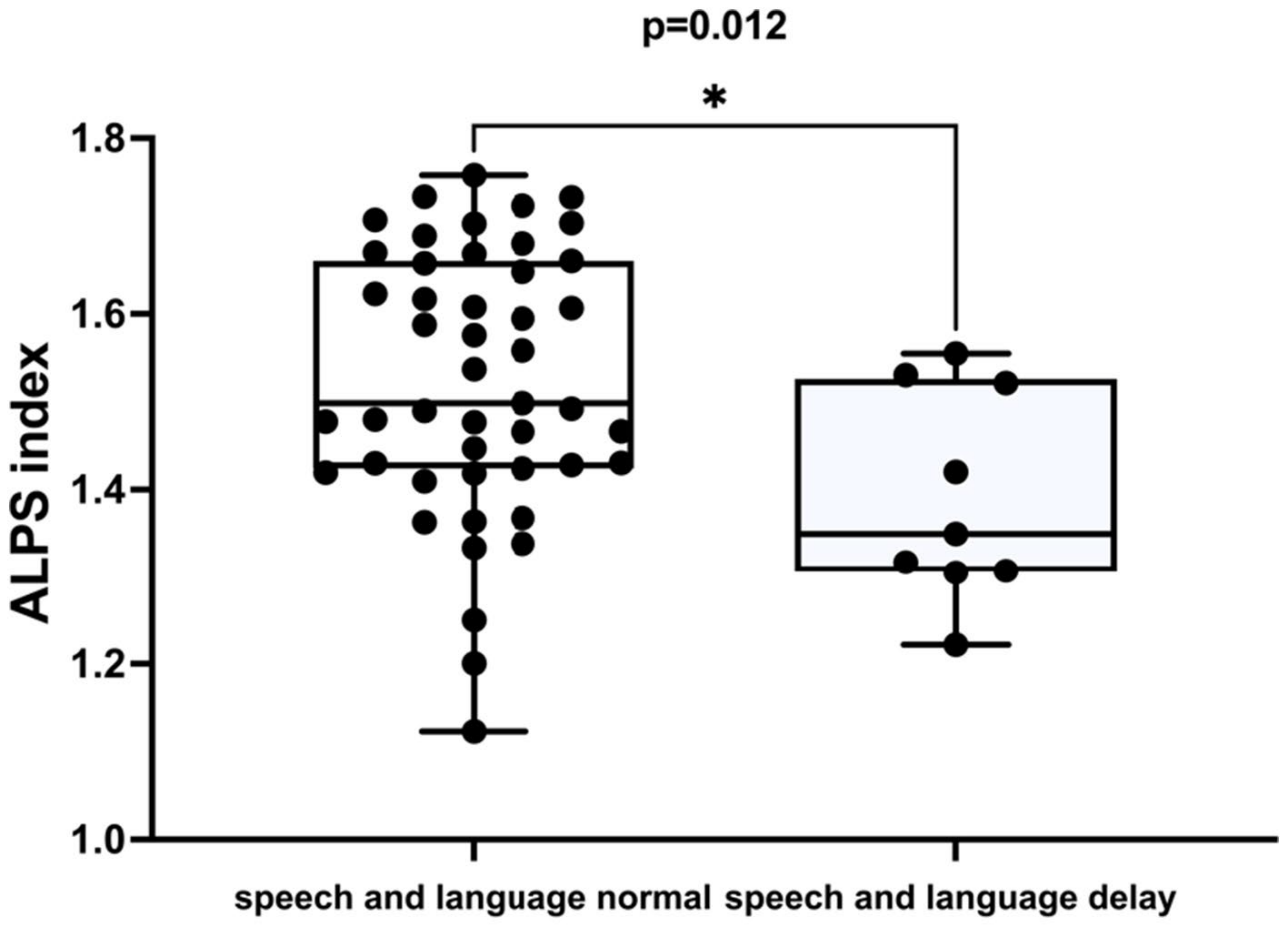
The difference in the ALPS index between individuals with normal speech and language development and those with delayed speech and language development in ADHD patients.

The DTI-ALPS index was negatively correlated with speech and language delay in ADHD (*r* = −0.335, *p* = 0.012) (Table 3; Figure 4). This correlation remained significant after adjusting for age and sex (*r* = −0.329, *p* = 0.015), as illustrated in Table 3. However, no significant correlation was observed between the DTI-ALPS index and gross motor retardation in ADHD patients.

**Table 3:** Correlation analysis of DTI-ALPS index with gross motor retardation and speech and language delay.

Variable		Unadjusted		Adjusted	
		Correlation r	*p* value	Partial correlation	*p* value
ALPS index	Gross motor retardation	−0.124	0.361	−0.133	0.338
	Speech and language delay	−0.335	0.012	−0.329	0.015

The partial correlation coefficients were adjusted for age and sex.

In order to test the reliability of retest, two radiologists plotted the region of interest of each subject, obtained the dispersion values of projection fibers and association fibers along x, y and z axes, and calculated the ALPS index. The inter-observer (ICC, 0.752–0.761) and intra-observer (ICC, 0.757–0.794) agreements were good.

## Discussion

The DTI-ALPS method is non-invasive, requires only a single scan, and does not require contrast agents. In the human brain, medullary arteries and veins run horizontally on cross-section. In a region proximal to the lateral ventricle, projection fibers, including the pyramidal tract, run in the vertical direction. On the outer side of the projection fibers, the commissural fibers, including the superior longitudinal fascicles, run in the anteroposterior direction. Thus, in this region, diffusion along the perivascular spaces of the medullary arteries and veins can be measured separately from the diffusion along large white matter fibers. In the DTI-ALPS technique, the diffusion capacity along the perivascular space in the white matter on the outer side of the body of the lateral ventricle is evaluated as a ratio of the diffusion capacity along the perivascular space to that in a direction perpendicular to the running direction of the main white matter fibers (ALPS index) (Taoka and Naganawa, 2020b). A recent study demonstrated that ALPS index is significantly correlated with the glymphatic clearance function detected in classical tracer studies (Zhang et al., 2021). Furthermore, excellent inter-observer agreements for the ALPS index have been reported (Bae et al., 2021). Thus, the ALPS index can be practically applied in human studies, providing a potential method to investigate glymphatic system function in various neurological diseases.

In this study, DTI-ALPS was employed to assess intracerebral glymphatic system activity and to evaluate the relationship between the glymphatic system and clinical scales, as well as gross motor and language development in ADHD patients. We found that the ALPS index in ADHD patients was significantly lower than that in the TD group, consistent with previous research suggesting impaired glymphatic system function in ADHD (Chen et al., 2024). Additionally, a negative correlation was observed between the ALPS index and Conners total scores in ADHD patients, indicating that glymphatic system dysfunction may be associated with the occurrence and development of ADHD. Further analysis revealed a negative correlation between the ALPS index and speech and language delay in ADHD patients.

Compared to age-matched TD children, ADHD patients exhibit significant difficulties in attention concentration, shorter attention spans, and excessive activity or impulsivity. The Conners Parent Symptom Questionnaire (PSQ) is a widely used tool for screening child behavior problems (Lv et al., 2023). Our study found a negative correlation between the ALPS index and Conners total scores in the ADHD group, suggesting that glymphatic system dysfunction may contribute to the pathogenesis of ADHD. This finding aligns with previous research (Chen et al., 2024). The biological mechanisms through which genetic and environmental factors act and interact to alter neurodevelopment in ADHD are not yet understood and there remains no diagnostic neurobiological marker (Thapar and Cooper, 2016). However, animal studies have implicated noradrenergic and dopaminergic neurotransmission, consistent with the neurochemical effects of ADHD medications, as well as serotonergic neurotransmission (Russell, 2011). One of the core symptoms of ADHD is hyperactivity, which is associated with excessive and extensive physical activity (Leitner et al., 2007). Physical activity is known to increase levels of norepinephrine and dopamine in the brain (Lin and Kuo, 2013). Norepinephrine acts as a primary neurotransmitter that suppresses the brain’s glymphatic system during wakefulness by inhibiting cerebrospinal fluid (CSF) flow in the choroid plexus (Jessen et al., 2015). This suppression results in weakened glymphatic fluid flow, which is a key manifestation of glymphatic system dysfunction. Moreover, the dopaminergic signaling is central to the regulation of arousal. Previous studies have shown that the activation of the glymphatic system is more enhanced during slow-wave sleep than during wakefulness (Taoka and Naganawa, 2020a; Xie et al., 2013). Sleep enhances the penetration of tracers into the brain parenchyma, facilitating the clearance of metabolic waste products (Xie et al., 2013; Eide et al., 2021). The pattern of neuronal activity during sleep is essential for cerebrospinal fluid to interstitial fluid (CSF-to-ISF) perfusion. During wakefulness, neurons fire in a highly desynchronized manner, maximizing information complexity for diverse cognitive tasks (Harris and Thiele, 2011), especially in children with ADHD. However, owing to out of-sync spiking, the field potentials generated by individual neurons cancel each other out, thereby producing only small fluctuations in the ISF. In contrast, neurons coordinate their actions to generate large-amplitude, rhythmic ionic oscillations in the ISF during sleep. These high-energy ionic waves facilitate the perfusion of fresh CSF through the parenchyma and the removal of metabolic waste products (Jiang-Xie et al., 2024). Therefore, the impairment of dopamine metabolism may lead to impairment in regulating sleep and wakefulness, which might be related to the glymphatic system function (Wynchank et al., 2017). In addition, disturbances in cerebrospinal fluid circulation can impair the clearance of harmful substances that accumulate in the brain and lead to neuroinflammation (Xie et al., 2013). While no single risk factor can fully explain ADHD, multiple genetic and environmental factors contribute to its development. The results of this study suggest that impaired glymphatic system function may be an important pathogenic mechanism underlying ADHD. Our study aligns with previous research (Zhang et al., 2024), supporting the central role of glymphatic system dysfunction in both neurodegenerative and neurodevelopmental disorders. The underlying mechanism may involve reduced CSF circulation efficiency, which impairs the clearance of metabolic waste products. In the context of ADHD, this could include neuroinflammatory mediators that accumulate due to insufficient glymphatic clearance, while in Alzheimer’s disease (AD), the key pathogenic metabolite is *β*-amyloid. Such impaired waste clearance disrupts the homeostatic balance of the brain microenvironment, ultimately affecting neural function. This shared pathological pathway highlights the glymphatic system as a critical node in maintaining brain health across different neurological conditions.

Children with autism have a higher rate of delayed motor development (Lloyd et al., 2013; Bowler et al., 2024). Early motor delays are also common in infants later diagnosed with ASD (Landa et al., 2013; Estes et al., 2015; Zwaigenbaum et al., 2009). Several previous reports in the pediatrics literature have reported an association between increased extra-axial EA-CSF and motor delays (Sahar, 1978; Nickel and Gallenstein, 1987; Lorch et al., 2004; Hellbusch, 2007). Shen et al. using both direct examination and parent-interviews of motor ability found that increased EA-CSF at 6 months was significantly associated with deficits in motor ability at 6 months in infants later diagnosed with ASD (Shen et al., 2017). This study aims to explore whether early motor delays in children with ADHD is related to glymphatic system dysfunction. However, no correlation was found, which may be due to the small sample size of ADHD patients in this study, moreover, motor and language development may depend on different neurobiological pathways. And motor skills involve a wider range of networks (such as the cerebellum and basal ganglia), and the ROI based on the lateral ventricle used in DTI-ALPS may fail to capture these networks. And the direct relationship between EA-CSF and glymphatic system needs to be further studied.

Several reviews have highlighted communication difficulties in ADHD. Geurts et al. (2010) published by 2010 concluded that these studies formed a small but consistent body of evidence that children with ADHD have pragmatic language impairments relative to typical children, though less severe than those with ASD. St Pourcain et al. (2011) also found that 82% of the children with persistent hyperactivity/inattention had social-communication difficulties. But so far, the mechanism of its occurrence has not been clearly clarified. This study revealed a negative correlation between the ALPS index and speech and language delay in ADHD patients, suggesting that language delay may share a common neuropathological basis with ADHD, namely impaired glymphatic system function. Recent studies have shown that CSF perfusion requires rhythmic neuronal activity for both entry into and clearance from the brain (Jiang-Xie et al., 2024). Previous studies have shown that healthy adults and children with typical language development exhibit greater grey and white matter density in the left hemisphere, which supports language function and shows left-greater-than-right asymmetry in white matter pathways connecting major language areas (Powell et al., 2006), This left hemisphere specialization begins early in development and becomes more pronounced as language complexity increases (Holland et al., 2007). Neurobiological maturational, developmental processes continue from childhood to adulthood (Friederici et al., 2012), and delayed language development may indicate fewer neurons in the corresponding brain parenchyma or a relative decrease in neuronal activity, resulting in impaired lymphatic clearance.

## Limitations

This is the first study to investigate glymphatic system function in children with ADHD and explore its relationship with language delay. However, several limitations should be acknowledged. First, the ALPS index only reflects the transient glymphatic function at the time of examination, and most subjects were scanned during wakefulness. Second, the study design was cross-sectional. And then, children’s language abilities are constantly evolving. Longitudinal studies are needed to fully characterize the developmental trajectory and glymphatic system function in children with ADHD. Additionally, the post-hoc power of 76% for the primary outcome (ALPS index) was slightly below 80%, indicating a possibility of Type II error. Future studies with larger sample sizes are recommended to confirm these findings. Moreover, although socioeconomic status was not formally measured, the homogeneous socioeconomic background of this urban cohort may have mitigated its confounding effect. Future studies should incorporate standardized socioeconomic status metrics for comprehensive control. And a limitation of the DTI-ALPS method is its reliance on ROI settings. Even though we placed the ROIs strictly in accordance with the literature, standardized automated ROI placement protocols are required in future studies to eliminate arbitrariness. Finally, the participants lacked magnetic susceptibility-weighted imaging (SWI) sequences, which are more sensitive for visualizing cerebral medullary veins compared to conventional MRI. Registering SWI sequences with DTI sequences would enhance the accuracy of region of interest delineation.

## Conclusion

Glymphatic system function appears to be impaired in ADHD patients, and this study is the first to demonstrate that speech and language delay in ADHD may be related to impaired glymphatic system function. This suggests that speech and language delays in ADHD comorbidities may share some of the same pathological mechanisms as ADHD itself. Early intervention may be linked to better language trajectories in ADHD, providing a rationale for longitudinal trials to test causality.

## Data Availability

The raw data supporting the conclusions of this article will be made available by the authors, without undue reservation.
